# Optimization of Sugar-Substituted Strawberry Preserve Formulation via Back-Propagation Neural Network

**DOI:** 10.3390/foods15183342

**Published:** 2026-09-20

**Authors:** Qing Gao, Nanxin Wang, Yutian Wang, Shuxin Ye, Jinsong He

**Affiliations:** College of Food Science and Technology, Yunnan Agricultural University, Kunming 650201, China; 2015053@ynau.edu.cn (Q.G.); 18812379482@163.com (N.W.); wytwd27@163.com (Y.W.); m13628691513@163.com (S.Y.)

**Keywords:** sugar substitute, strawberry preserve, Box–Behnken response surface methodology, back-propagation neural network, xylitol, isomaltooligosaccharide

## Abstract

Single-factor experiments were conducted with sensory score set as the evaluation index to investigate individual effects of sugar substitution dosage, mass ratio of xylitol (Xyl) to isomaltooligosaccharide (IMO), and osmotic soaking duration on the comprehensive quality of low-sugar strawberry preserves. Box–Behnken design (BBD)-based response surface methodology (RSM) and back-propagation neural network (BPNN) models were subsequently established for parallel process optimization and comparative modeling analysis based on single-factor experimental datasets. Physicochemical indicators including total sugar, titratable acidity, total flavonoids, total phenolics, chromatic aberration, moisture content and water activity of low-sugar strawberry preserves manufactured under optimized technological parameters were determined and systematically compared with those of sucrose-controlled counterparts. The RSM and BPNN models predicted maximum sensory scores of 81.62 and 82.62, respectively. Experimental verification conducted under the practically adjusted optimal conditions derived from the RSM and BPNN models yielded sensory scores of 81.63 and 82.72, respectively, with the BPNN-derived condition achieving a higher verified sensory score than the RSM-derived condition. Based on these results, the optimal processing parameters were determined as a 40% (*w*/*w*) addition amount of compound sugar substitute, a Xyl/IMO mass ratio of 1.5, and a soaking time of 5.5 h. Lower total sugar and titratable acidity, yet higher contents of total phenolics and flavonoids, were detected in sugar-substituted strawberry preserves in comparison with sucrose-preserved samples. Meanwhile, stronger red–yellow chromaticity was observed in sucrose-based preserves, while sugar-substituted products delivered higher lightness. Superior predictive capacity of the BPNN model to RSM was validated in the formulation optimization of strawberry preserves. Our experimental findings show that partially replacing sucrose with functional sweeteners improves the nutritional attributes of fruit preserves without impairing their sensory acceptability.

## 1. Introduction

*Fragaria × ananassa* Duch., namely strawberry, is a widely consumed fruit abundant in bioactive constituents including vitamin C, phenolic substances and flavonoids, whose attractive red pigmentation renders high market acceptance [1]. Nevertheless, thin epidermis and high moisture content ranging from 90% to 95% are inherent properties of strawberries, so severe mechanical damage and microbial contamination are easily incurred after harvesting, accelerating fruit deterioration and spoilage; consequently, merely 1–2 days of shelf life can be maintained for fresh strawberries under ambient conditions [2]. Transformation of fresh strawberries into preserved fruit products such as candied strawberries is therefore recognized as an important technical approach for improving their processing characteristics and overall product quality. Fruit preserves represent a traditional category of Chinese fruit deep-processing products, whose production generally involves sugar infiltration and hot-air drying procedures to obtain desirable sensory and physicochemical properties. Conventional strawberry preserves are commonly sweetened with sucrose via osmotic dehydration and sulfitation treatments, leading to final products with total sugar concentrations reaching 60–70% [3]. Excessive sucrose consumption has been closely associated with dental caries [4]. Growing consumer demand for healthy food has driven low-sugar, sulfite-free fruit preserves retaining natural fruit flavor to become a dominant developmental trend in the fruit processing industry. Functional sweeteners are thus widely adopted for partial sucrose substitution to enhance the quality of low-sugar preserves. IMO exhibits prebiotic properties that boost the proliferation of beneficial intestinal bifidobacteria and resist gastrointestinal digestion in humans [5]. Xyl has a relatively low caloric value and an insulin-independent metabolism, while eliciting lower glycemic and insulinemic responses than sucrose, making it a promising sugar substitute for individuals concerned about glycemic control [6].

In recent years, BPNN algorithms have attracted considerable research attention owing to their outstanding nonlinear mapping capabilities. BPNNs are multilayer feed-forward neural networks generally consisting of an input layer, one or more hidden layers, and an output layer, in which network parameters are continuously adjusted through error back-propagation to establish nonlinear relationships between processing variables and target responses. This data-driven modeling capability is particularly suitable for food processing systems involving complex interactions among variables. Compared with conventional statistical methods, BPNNs can improve the modeling and prediction accuracy of complex food processing systems while compensating for the limitations of RSM in addressing highly nonlinear relationships among variables [7]. Li et al. and Zhang et al. adopted combined RSM-BPNN modeling to optimize extraction processes and demonstrated that BPNNs deliver higher predictive accuracy than standalone RSM, providing more reliable parameter guidance for technological optimization [8,9]. Joint application of these two modeling methods therefore enables more precise optimization of formulation and processing parameters for low-sugar strawberry preserves. Related studies have explored sweetener-based formulation optimization for confectionery products such as sucrose-free hard candy [10].

Unlike prior RSM-BPNN studies focused on plant bioactive-component extraction and sucrose-free hard-candy research lacking systematic multi-index physicochemical and bioactive-substance comparisons against sucrose-based controls, the present work employs the combined RSM-BPNN strategy for sugar-reduction optimization of strawberry preserves with unique osmotic-soaking processing and further compares finished-product quality between sugar-substituted and sucrose-control groups.

The fruit-processing industry urgently needs strawberry preserves that meet consumer health demands while maintaining favorable sensory profiles. Partially replacing sucrose with functional sweeteners such as IMO and Xyl offers an effective strategy to reduce health risks caused by conventional high-sugar fruit preserves. In this study, we used single-factor experiments combined with RSM and BPNN modeling to screen the optimal formulation and processing parameters for low-sugar strawberry preserves. We also performed comprehensive physicochemical characterization of product quality to provide theoretical bases and technical references for the industrial development of low-sugar strawberry preserves.

## 2. Materials and Methods

### 2.1. Experimental Materials and Instruments

#### 2.1.1. Raw Materials and Chemical Reagents

Fresh strawberries (*Fragaria × ananassa* Duch.) were purchased from Hema Fresh Supermarket, China. Food-grade IMO was supplied by Shandong Chuangyuan Biotechnology Co., Ltd. (Jinan, Shandong, China), and food-grade Xyl was obtained from Shandong Futian Pharmaceutical Co., Ltd. (Jinan, Shandong, China). Food-grade carrageenan was provided by Linyi Aidisen Biotechnology Co., Ltd. (Linyi, Shandong, China); food-grade sucrose was purchased from Weixianyuan Co., Ltd. (Jinan, Shandong, China); food-grade citric acid was supplied by Shandong Yingxuan Industrial Co., Ltd. (Jinan, Shandong, China); and food-grade carmine was obtained from Shandong Bailong Biotechnology Co., Ltd. (Jinan, Shandong, China). Analytical-grade phenol was sourced from Macklin Biochemical Co., Ltd. (Shanghai, China). Analytical-grade glucose, sodium hydroxide, sodium carbonate, sodium nitrite and aluminum nitrate were all purchased from Shanghai Aladdin Biochemical Technology Co., Ltd. (Shanghai, China). Concentrated sulfuric acid and anhydrous ethanol of analytical grade were supplied by Guangdong Guanghua Sci-Tech Co., Ltd. (Shantou, Guangdong, China), and analytical-grade phenolphthalein was obtained from Chengdu Changlian Chemical Reagent Co., Ltd. (Chengdu, Sichuan, China). Folin–Ciocalteu reagent and gallic acid (purity ≥ 98%) were provided by Hengchao Biotechnology Co., Ltd. (Nanjing, Jiangsu, China), and rutin standard substance (purity > 99%) was purchased from Dr Ehrenstorfer GmbH (Augsburg, Germany).

#### 2.1.2. Experimental Instruments

HH-8 thermostatic water bath was manufactured by Chengxi Zhengrong Experimental Instrument Factory (Changzhou, Jiangsu, China). SK3200HP ultrasonic cleaning apparatus was produced by Shanghai Keda Ultrasonic Co., Ltd. (Shanghai, China) and operated at a frequency of 53 kHz. TTL-10B ultrapure water system (Beijing Tongtailian Technology Co., Ltd., Beijing, China) was applied for ultrapure water preparation. V-5600 UV–Vis spectrophotometer (Shanghai Yuanxi Instruments Co., Ltd., Shanghai, China) was used for ultraviolet-visible absorbance measurement. HC-2062 high-speed centrifuge (Anhui Zhongke Zhongjia Technology Co., Ltd., Hefei, Anhui, China) was used for sample centrifugation. CM-5 spectrophotometer (Konica Minolta, Tokyo, Japan) was utilized for colorimetric measurement. GZX-GF101-8-B electrothermal constant-temperature drying oven (Shanghai Yuejin Medical Instruments Co., Ltd., Shanghai, China) was employed for sample dehydration. HD-3A intelligent water activity meter (Huake Instrument & Equipment Co., Ltd., Nanjing, Jiangsu, China) and HS-153 moisture analyzer (Shanghai Youyi Instruments Co., Ltd., Shanghai, China) were separately applied for water activity and moisture content determination.

### 2.2. Production Technological Route of Strawberry Preserves

The standardized manufacturing workflow of strawberry preserves was arranged as strawberry cleaning → calyx removal → hot blanching → draining treatment → ultrasonic-assisted osmotic sugar permeation → thermostatic sugar impregnation → hot-air drying → moisture balance tempering → sealed packaging.

### 2.3. Core Technological Operations

Fresh strawberries with 80–90% ripeness, free from disease spots and mechanical damage, were selected as raw materials, followed by stem and sepal removal, thorough cleaning and 1–2 min boiling-water blanching. Compound sugar substitutes consisting of Xyl and IMO together with sucrose were weighed proportionally based on strawberry mass, and 0.4% carrageenan, 0.5% citric acid and 0.1% carmine were incorporated into the mixed sugar system. Ultrasonic-assisted osmotic treatment was conducted under 150 W ultrasonic power and 45 °C for 66 min prior to sugar impregnation in a thermostatic water bath maintained at 55 °C. Excess syrup was drained off from strawberry samples after impregnation, and subsequent drying was performed at 65 °C for 10 h before tempering treatment for uniform moisture distribution. Composite plastic packaging materials were finally adopted for airtight encapsulation of finished strawberry preserves [11].

### 2.4. Single-Factor Experiments

Single-factor trials were carried out with sensory score set as the response variable to investigate separate influences of sweetener addition dosage (30%, 35%, 40%, 45%, 50% of strawberry mass), Xyl/IMO mass ratios (1:2, 1:1, 3:2, 2:1, 5:2) and osmotic soaking duration (4 h, 5 h, 6 h, 7 h, 8 h). Primary optimal dosage ranges of the three technological factors were screened from single-factor experimental outcomes for subsequent formulation and process optimization of strawberry preserves. Each treatment level was performed in three independent production batches (*n* = 3), and sensory evaluation was implemented for every batch.

### 2.5. Box–Behnken Response Surface Design

A three-factor, three-level BBD was adopted for parametric optimization of processing technologies, with sweetener addition amount (A), Xyl/IMO mass ratio (B) and soaking time (C) defined as independent variables and sensory score (Y) assigned as the response value. The experimental matrix was constructed according to screened factor levels as presented in Table 1.

### 2.6. Sensory Evaluation

The sensory scoring criteria for each evaluation index were formulated in accordance with sensory assessment protocols described by Liu et al. [12], and the detailed scoring standards are illustrated in Table 2. Ten trained panelists were organized for blind sensory evaluation of strawberry preserve samples following unified scoring rules, and the average values of all panel scores were calculated as final sensory results.

### 2.7. Construction of Back-Propagation Neural Network Model

A BPNN model was established on the basis of BBD experimental datasets to realize parallel comparison of predictive precision and optimization capacity between BPNN and RSM, and the reliability of optimized technological parameters was further verified simultaneously. Nonlinear mapping correlations between processing variables and sensory quality were characterized via the constructed network model, and sensory scores corresponding to non-sampled points within the experimental parameter space were predicted accordingly. Optimal processing conditions were confirmed through comparative analysis of predictive results generated by BPNN and RSM models. All 17 groups of BBD experimental data were adopted as network training datasets, with sweetener addition dosage, Xyl/IMO ratio and soaking time selected as input variables and sensory score defined as the single output variable. MATLAB software (R2024b, MathWorks, Natick, MA, USA) was utilized for network construction. The initial neuron quantity of the hidden layer was set to a range of 3–13 according to the Kolmogorov–Arnold theorem [13]. The total dataset was randomly divided into training subsets (70%), verification subsets (15%) and test subsets (15%). Model performance was assessed via mean square error (MSE), mean absolute error (MAE) and correlation coefficient (R) between predicted and measured sensory values. Iterative adjustment of network topology and training hyperparameters was implemented to minimize MAE and MSE while maximizing correlation coefficients, through which the optimal BPNN structure was obtained.

### 2.8. Determination of Physicochemical Indicators

Parallel parametric optimization was completed via RSM and BPNN models. Multiple physicochemical indices, including total sugar, titratable acidity, total flavonoids, total phenolics, chromatic aberration, moisture content, and water activity of low-sugar strawberry preserves manufactured under optimized technological parameters were quantified and compared with those of sucrose-group control samples.

#### 2.8.1. Determination of Total Sugar Content

The phenol–sulfuric acid colorimetric method was adopted for total sugar measurement [14]. Serial concentration gradients of glucose standard solution were prepared, followed by sequential addition of phenol reagent and concentrated sulfuric acid for chromogenic reaction. After sufficient mixing and cooling to ambient temperature, absorbance values were detected at 490 nm with a 50% ethanol solution set as the blank control. A standard calibration curve was plotted with glucose concentration as the abscissa and absorbance as the ordinate, generating a linear regression equation of *y* = 1.6382*x* + 0.1940 and a determination coefficient (*R*^2^) of 0.9941.

#### 2.8.2. Determination of Titratable Acidity

The acid-base indicator titration method specified in the national food safety standard GB 12456-2021 was applied for titratable acidity measurement [15]. A total of 10 g homogenized sample was diluted to a constant volume with distilled water and filtered to acquire test solution. A certain volume of filtrate was transferred into an Erlenmeyer flask and diluted with distilled water, followed by addition of 2–4 drops of phenolphthalein indicator. Titration with 0.1 mol/L NaOH standard solution was conducted until a persistent faint pink color was maintained for 30 s, and the consumed volume of NaOH solution was recorded. A blank test using distilled water instead of the sample was performed for systematic error correction. Titratable acidity was calculated as malic acid equivalents using the following equation:

(1) $$(g/kg)=(V-V_{0})\times c\times M\times F/m$$

where V is the volume of NaOH solution consumed by the sample (mL), V_0_ is the volume consumed in the blank test (mL), c is the concentration of NaOH (mol/L), M is the molar mass of malic acid (134.09 g/mol), F is the dilution factor, and m is the sample mass (g).

#### 2.8.3. Determination of Total Flavonoid Content

The rutin colorimetric assay reported in the literature [16,17] was adopted for total flavonoid quantification. Serial diluted solutions of rutin standard substance were prepared, and the chromogenic reaction was implemented via a sodium nitrite–aluminum nitrate–sodium hydroxide composite system. Absorbance values were detected at 510 nm after complete color development for standard curve plotting. A linear regression equation of *y* = 0.1700*x* + 0.05841 with an *R*^2^ value of 0.9830 was acquired.

#### 2.8.4. Determination of Total Phenolic Content

The Folin–Ciocalteu colorimetric method with gallic acid as the standard substance was adopted for total phenolic measurement [18]. Precisely prepared serial dilutions of gallic acid standard solution were transferred into test tubes, followed by sequential addition of Folin–Ciocalteu reagent and 7.5% Na_2_CO_3_ solution. Reaction systems were diluted to a constant volume of 10 mL and incubated in dark environments for 1 h before absorbance detection at 765 nm. A standard calibration curve was plotted to obtain the regression equation *y* = 1.1122*x* − 0.0275 and an *R*^2^ value of 0.9994.

#### 2.8.5. Determination of Chromatic Aberration

Strawberry preserve samples with uniform particle size were selected, and surface color parameters at the fruit’s central regions were measured by a spectrophotometer to acquire CIE L*, a* and b* values. Three parallel measurements were conducted for each sample, and average color parameters were recorded. Total chromatic aberration (∆E) was calculated via the following formula [19]:

(2) $$∆E=\sqrt{{\left(L_{0}^{*}-L^{*}\right)}^{2}+{\left(a_{0}^{*}-a^{*}\right)}^{2}+{\left(b_{0}^{*}-b^{*}\right)}^{2}}$$

where ∆E represents total chromatic aberration; L*, a* and b* denote color parameters of strawberry preserve samples; L_0_*, a_0_* and b_0_* stand for corresponding color parameters of fresh strawberry raw materials.

#### 2.8.6. Determination of Moisture Content and Water Activity

Moisture content of strawberry preserves was directly quantified via a moisture analyzer. Water activity (*a*_w) was determined by the diffusion method with a water activity meter following the national standard GB 5009.238–2016 Determination of Water Activity in Foods [20].

### 2.9. Data Statistical Analysis

All raw experimental data were sorted and calculated with Microsoft Excel software. MATLAB (R2024b, MathWorks, Natick, MA, USA) was utilized for BPNN model construction. Origin 2024 software was adopted for all graphical plotting. Statistical significance analysis, including an independent-samples *t*-test, was completed via SPSS 23.0 software, and Design-Expert 12.0 software was applied for response surface parametric optimization.

## 3. Results and Discussion

### 3.1. Individual Effects of Single Technological Factors on Sensory Quality of Strawberry Preserves

#### 3.1.1. Influence of Xyl/IMO Mass Ratio on Sensory Score

**Figure 1:**
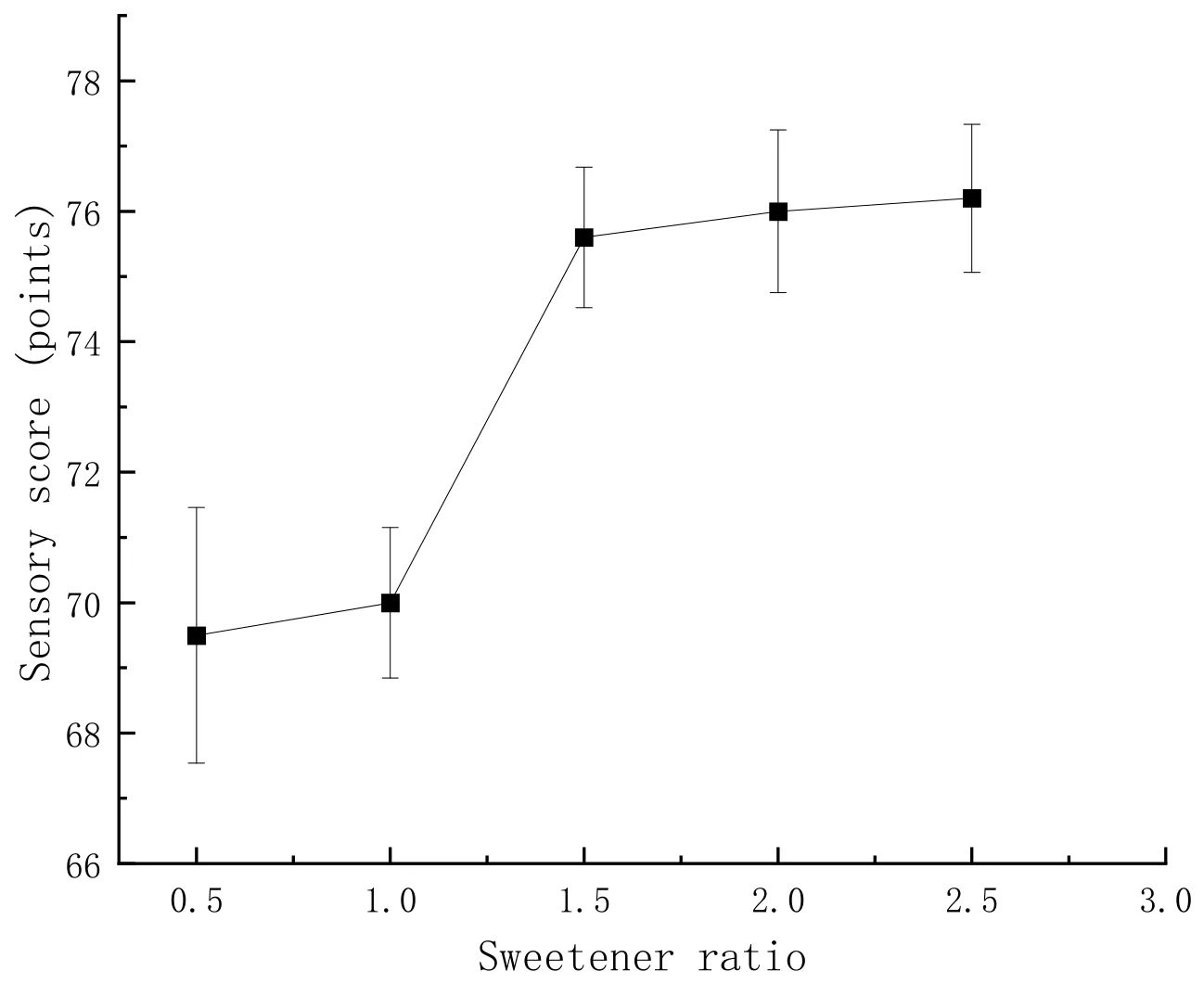
Effect of different sugar substitute ratios on the sensory scores of strawberry preserves (*n* = 3).

An evaluation of sensory scores corresponding to varying Xyl/IMO ratios was implemented under a fixed sugar substitute dosage (40% strawberry mass) and soaking time (5 h), and experimental outcomes are displayed in Figure 1. Relatively stable sensory scores were observed at low Xyl proportions, followed by rapid score elevation and subsequent plateauing with increased Xyl ratios, and a peak sensory score of 75.67 was obtained at the turning point ratio of 1.5.

The compound sugar–substitute ratio directly regulates syrup viscosity and penetration uniformity, further modifying the texture of final preserves. Elevated IMO proportion leads to increased syrup viscosity and weakened penetration homogeneity, resulting in a sticky fruit texture [21]. Meanwhile, insufficient xylitol addition produces a noticeable sour-astringent taste and reduces overall sensory acceptability [10]. Continuous elevation of Xyl proportion improves preserve texture accompanied by sharp sensory score growth, yet excessive xylitol dosage speeds up syrup infiltration, producing hard fruit texture and overly sweet notes and worsening overall sensory quality [22]. Higher Xyl/IMO ratios were therefore excluded from subsequent optimization experiments. Three gradient levels of 1.0, 1.5 and 2.0 were selected to cover critical variable ranges around the response turning point for reliable response surface design.

#### 3.1.2. Influence of Sugar Substitute Addition Dosage on Sensory Score

**Figure 2:**
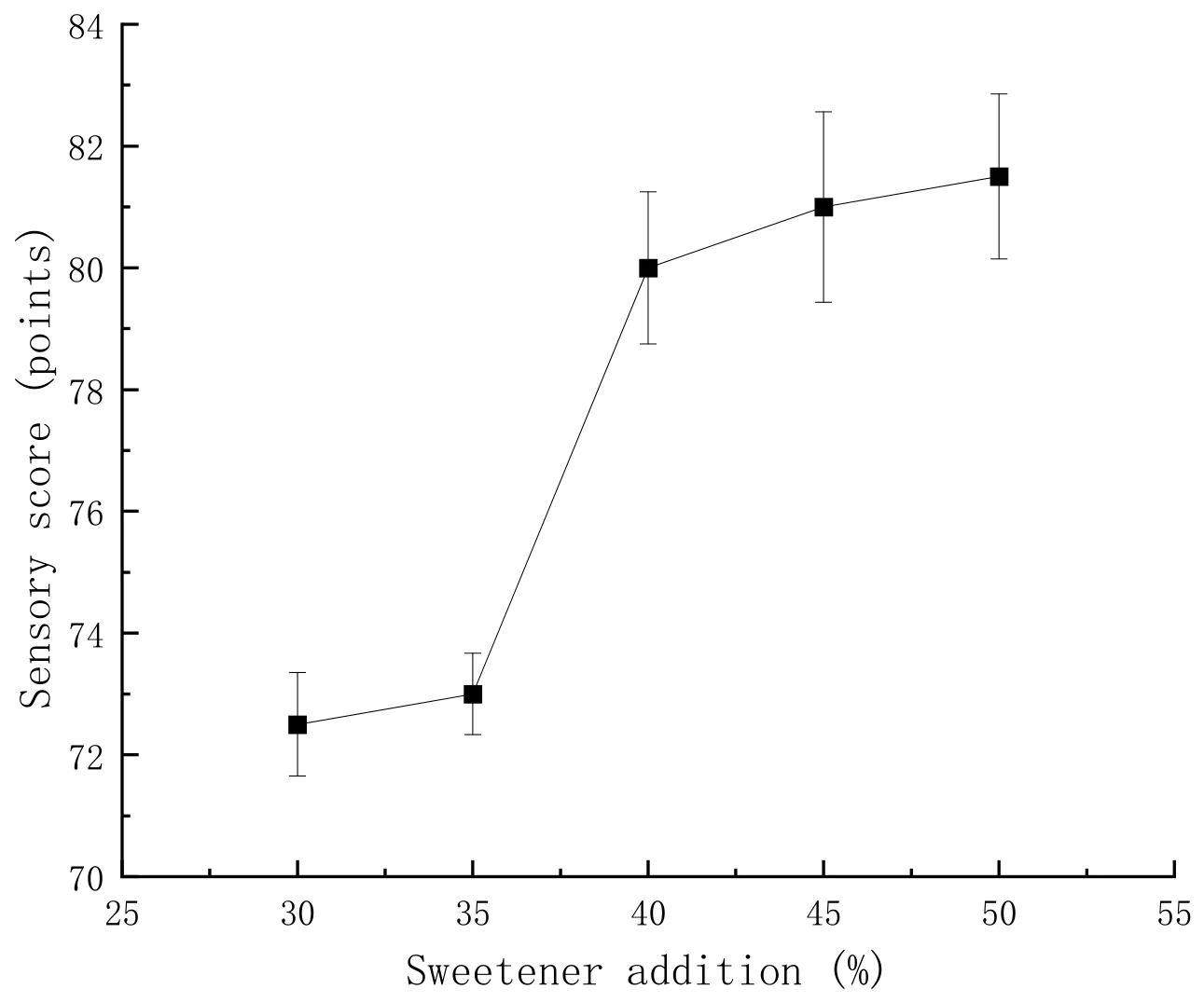
Effect of different sugar substitute addition levels on the sensory scores of strawberry preserves (*n* = 3).

The impacts of sugar substitute addition dosage on sensory scores were investigated under a fixed Xyl/IMO ratio of 3:2 (*w*/*w*) and soaking time of 5 h, as illustrated in Figure 2. Gradual growth of sensory scores was detected with increased substitute dosage; an inflection point was observed at 40% addition, where the sensory score reached approximately 80.0. Further increases in sugar-substitute dosage resulted in only limited additional improvement in sensory score, indicating a gradual plateauing trend rather than a sharp increase.

The total dosage of compound sugar substitutes governs the sweet–sour balance of strawberry preserves. Insufficient sweetener addition cannot neutralize the intrinsic sourness of strawberry pulp and yields a strong sour taste and poor sensory performance. Optimal sweet-acid equilibrium is achieved at a 40% addition dosage to generate peak sensory scores, whereas further dosage elevation induces excessive sweetness to mask natural strawberry flavor alongside increased raw material costs, so higher addition levels were eliminated from the optimization design. Three gradient levels of 35%, 40% and 45% were screened to cover response ranges of insufficient neutralization and over-sweetness for subsequent response surface analysis.

#### 3.1.3. Influence of Osmotic Soaking Time on Sensory Score

**Figure 3:**
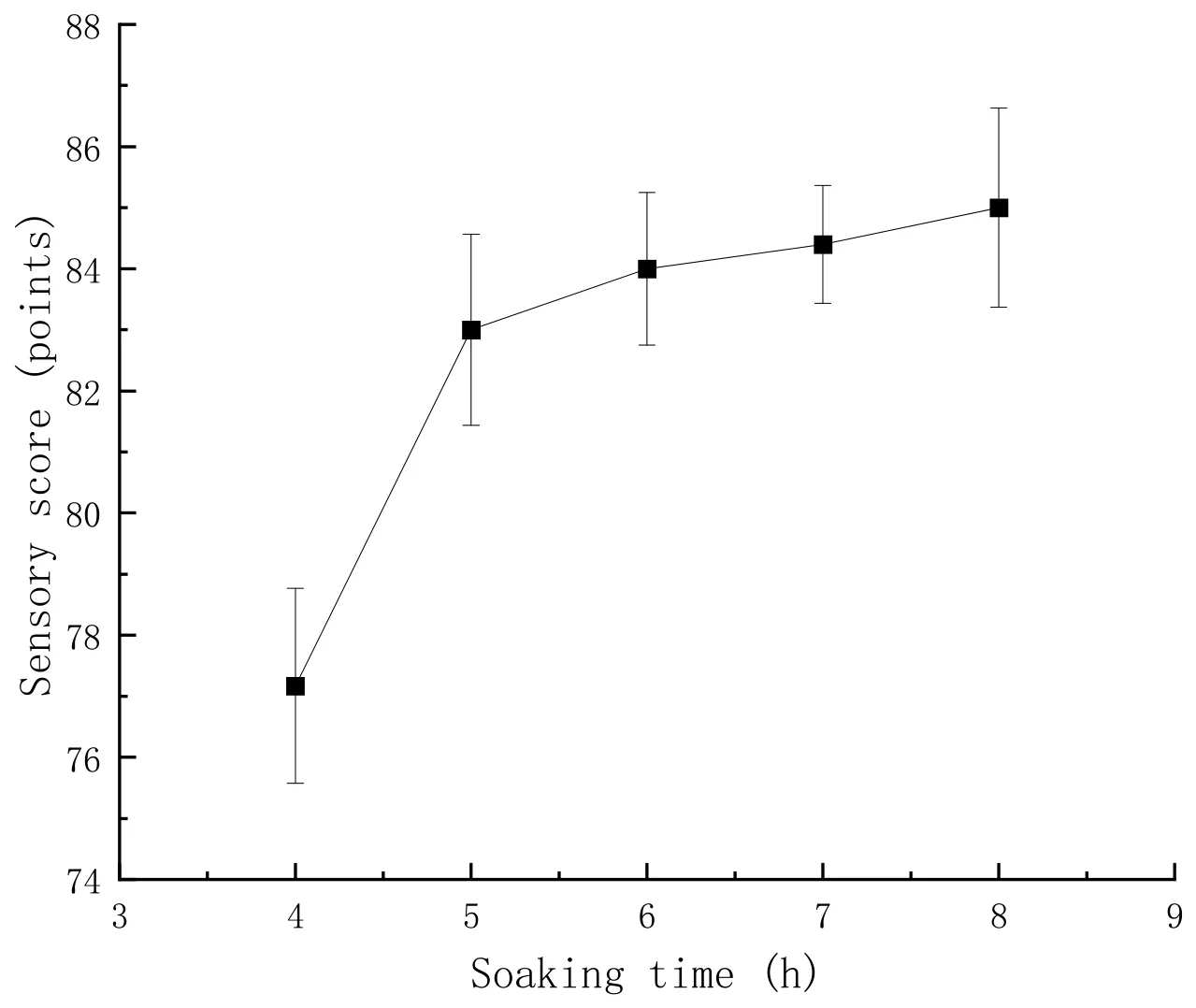
Effect of different soaking times on the sensory scores of strawberry preserves. (*n* = 3).

Variations in sensory scores corresponding to different soaking durations were measured under fixed sugar substitute dosage (40%, *w*/*w*) and Xyl/IMO ratio (3:2, *w*/*w*), and the results are presented in Figure 3. Rapid sensory score growth from 77.17 to 83.00 was recorded when soaking time was prolonged from 4 h to 5 h, indicating an obvious response inflection point, while only slight promotion of sensory quality was detected with soaking duration exceeding 5 h to form a stable response platform.

Such response variation can be explained by mass transfer kinetics during osmotic sugar permeation. Insufficient sugar infiltration and uneven internal syrup distribution are induced by a short soaking duration of 4 h, whereas a near-equilibrium state of internal–external sugar diffusion is realized at 5 h soaking time to optimize fruit texture and flavor balance. Limited additional mass transfer improvement is generated by further prolonged soaking time alongside elevated processing duration and energy consumption. Three gradient soaking durations of 4 h, 5 h and 6 h were ultimately selected to cover critical ranges of inadequate and excessive sugar permeation for subsequent RSM optimization.

### 3.2. Response Surface Modeling and Result Analysis

#### 3.2.1. Bbd Parametric Optimization

A response surface experimental design was constructed in accordance with BBD fundamental principles and single-factor screening outcomes based on factor levels listed in Table 1, and the complete experimental matrix together with corresponding response values are summarized in Table 3.

#### 3.2.2. Regression Model Establishment and Analysis of Variance

Quadratic regression fitting was conducted via response surface statistical software to acquire the regression equation describing the quantitative correlation between sensory score (Y) and independent variables A, B and C:

*Y* = 80.67 + 1.45*A* + 1.06*B* + 1.31*C* + 0.1250*AB* − 0.0825*AC* + 0.6650*BC* − 0.8968*A*^2^ − 1.73*B*^2^ − 1.94*C*^2^

Analysis-of-variance results in Table 4 verify the highly significant overall performance of this model (*p* < 0.01). The non-significant lack-of-fit term with an F-value of 0.5277 and a *p*-value of 0.6867 (*p* > 0.05) confirmed the high reliability of the constructed regression model. The determination coefficient (*R*^2^) of 0.9742 and the adjusted determination coefficient (*R*^2^_Adj) of 0.9411 further validated an excellent fitting degree between the regression model and experimental data, through which reliable prediction and quantitative analysis of factor effects on sensory scores of strawberry preserves can be realized. Linear terms A, B, C and quadratic terms B^2^, C^2^ exert highly significant influences on sensory scores; while significant effects were detected for quadratic term *A*^2^ and interaction term *BC*, no statistical significance was identified for the remaining regression terms. Sequential factor importance, sorted by F-values, was determined as sugar substitute addition dosage (A) > soaking time (C) > Xyl/IMO mass ratio (B).

#### 3.2.3. Interaction Analysis of Response Surface Variables

**Figure 4:**
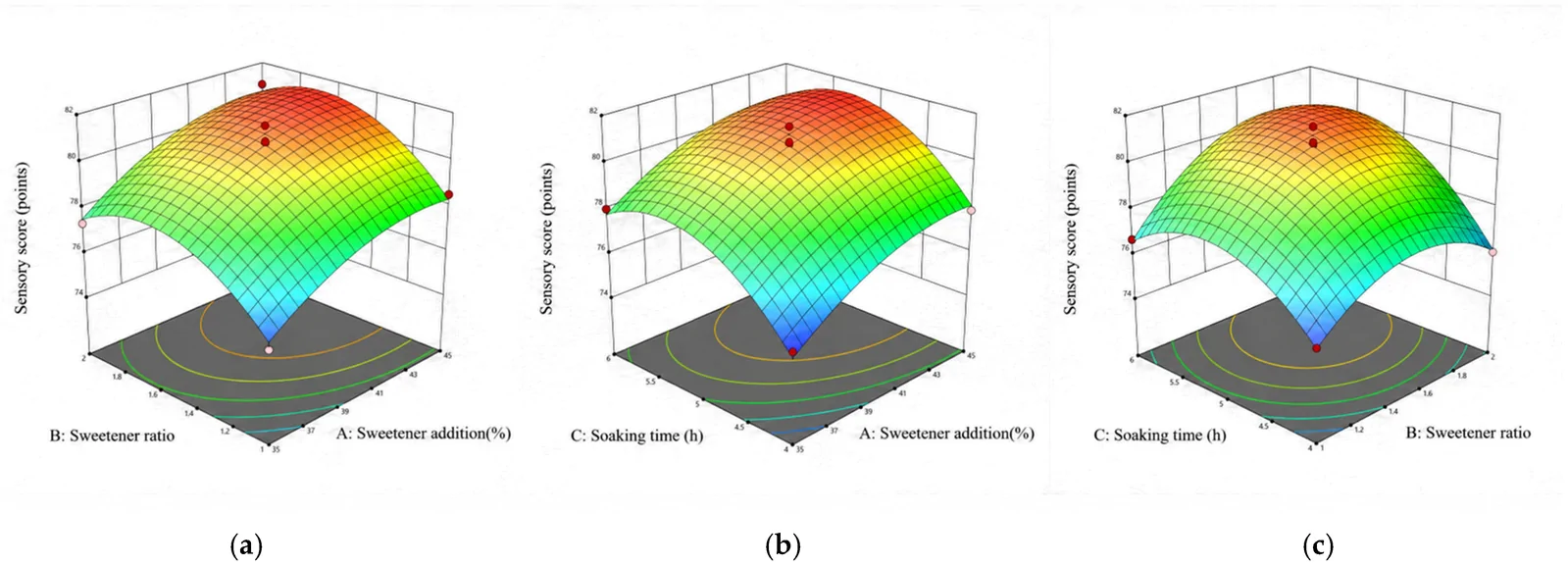
Three-dimensional response surface plots illustrating the interaction effects of independent variables on the sensory score of strawberry preserves. (The color gradient represents the sensory score, where red indicates a higher sensory score and blue indicates a lower sensory score. (**a**): Interaction effect of sweetener addition and sweetener ratio on sensory score; (**b**): Interaction effect of sweetener addition and soaking time on sensory score; (**c**): Interaction effect of sweetener ratio and soaking time on sensory score).

Three-dimensional response surface diagrams corresponding to sensory response values were generated via Design-Expert 12.0 statistical software to visualize interactive effects of sugar substitute dosage, Xyl/IMO ratio and soaking time on sensory scores, and the graphical results are displayed in Figure 4.

Both response-surface contour plots and ANOVA results validate the significant interaction between factor B and factor C. Under a fixed Xyl/IMO ratio, sensory scores increased initially and decreased subsequently with prolonged soaking duration; similarly, rising-falling variation trends of sensory scores were observed with elevated Xyl/IMO ratios under constant soaking time. Elliptical contour lines of the BC interaction group further verified prominent mutual effects between the two variables. Although curved surface morphology was exhibited by AB and AC interaction diagrams, no statistical significance was proven for corresponding interaction terms via variance analysis, which can be attributed to two primary causes. Firstly, surface curvature was mainly generated by significant quadratic terms of independent variables to reflect intrinsic nonlinear correlations between single factors and sensory scores rather than mutual interaction effects. Secondly, weak interaction intensities of AB and AC failed to reach the statistical significance threshold, so a comprehensive combination of variance analysis data and surface graphics is required for accurate result interpretation [23].

The optimized parametric combination predicted by the regression model was identified as 43.94% sugar substitute addition dosage, Xyl/IMO ratio of 1.58 and soaking time of 5.44 h, with a predicted sensory score of 81.62. Adjustment of theoretical optimal parameters to 45% substitute dosage, 1.5 Xyl/IMO ratio and 5.5 h soaking time was implemented for subsequent verification experiments considering practical industrial operability.

### 3.3. BPNN Modeling and Simulation Results

#### 3.3.1. Network Training and Performance Evaluation

**Figure 5:**
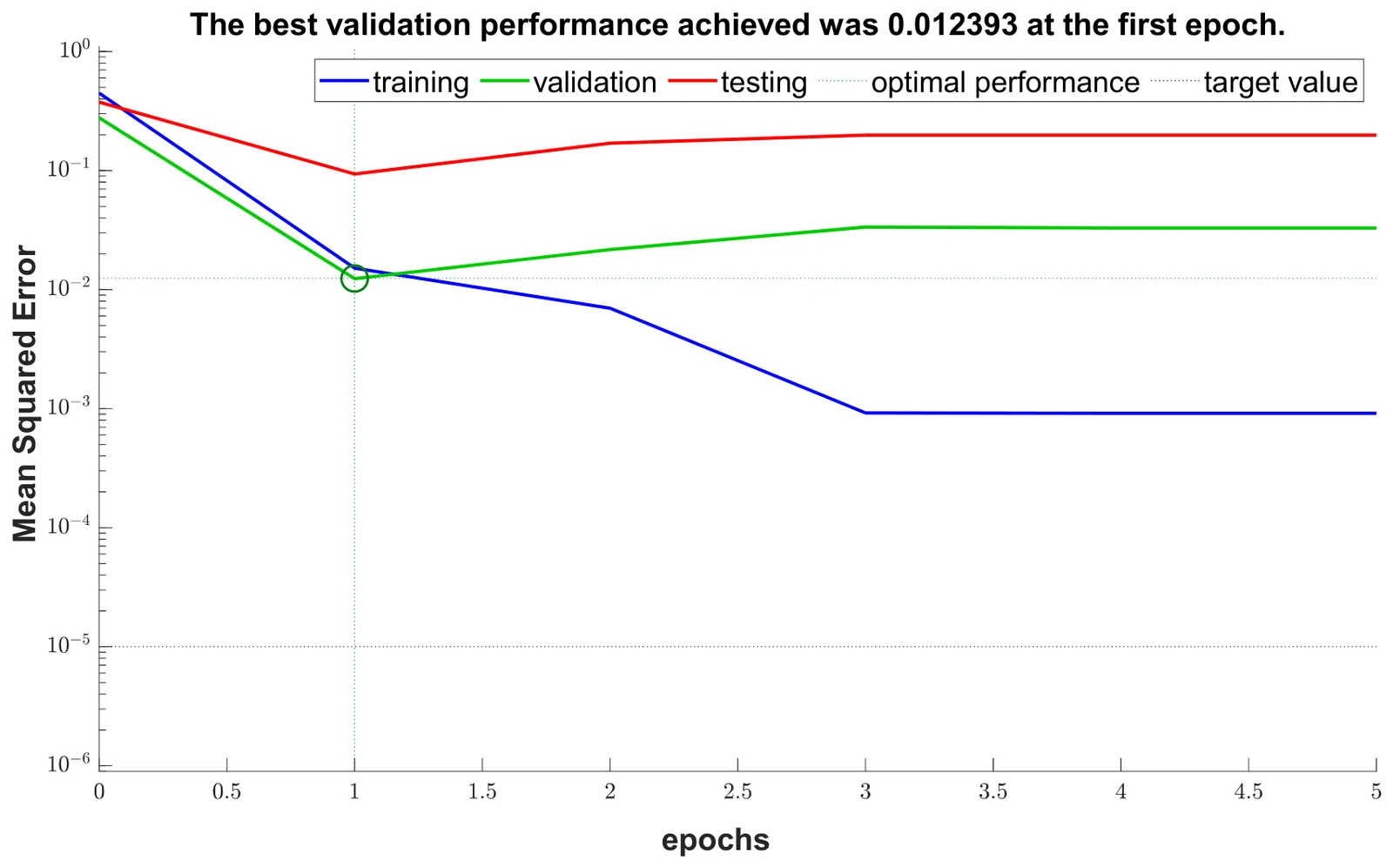
Training process of the back-propagation (BP) neural network model.

**Figure 6:**
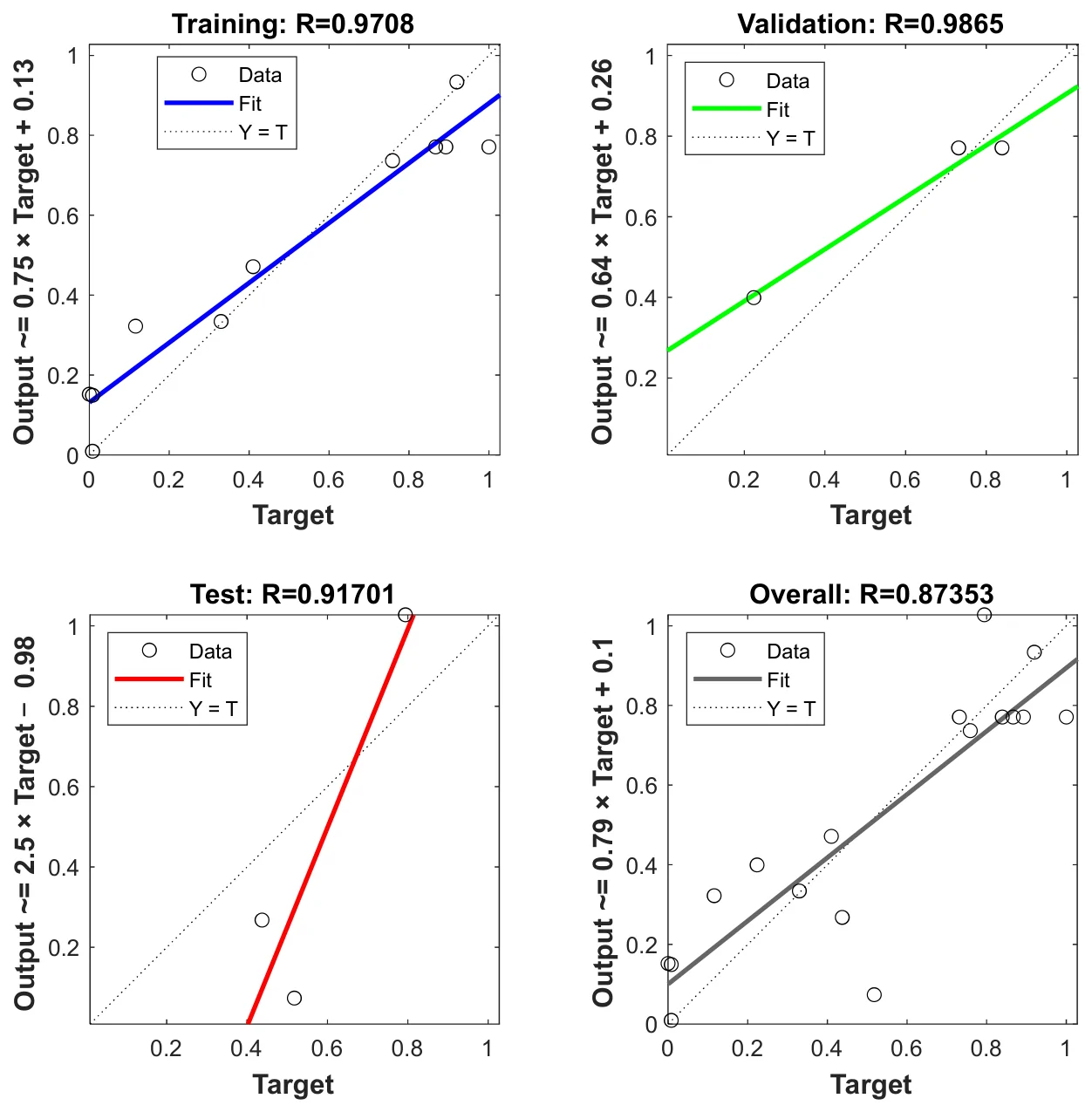
Regression performance of the BP neural network training results.

**Figure 7:**
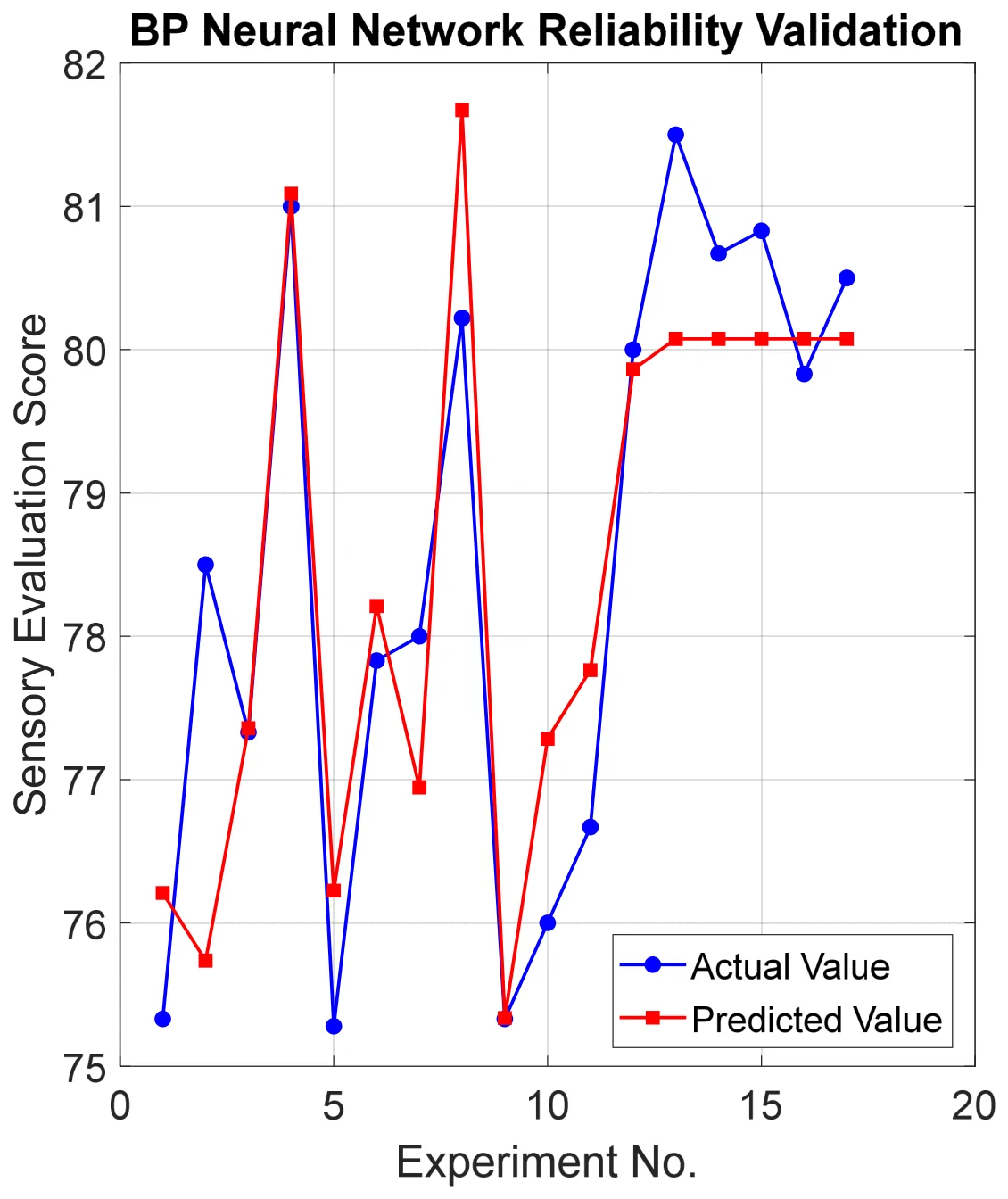
Validation of the reliability of the BP neural network model.

A 3–7–1 topological BPNN model was constructed for simulation and optimization of strawberry preserve processing parameters, and complete network training curves are presented in Figure 5. Lower MSE values correspond to higher data fitting precision of neural network models [24]. Optimal model verification performance was achieved at the first training epoch with a minimum MSE of 0.01239. Regression fitting diagrams of training datasets are illustrated in Figure 6, with correlation coefficients (R) of 0.9708 for training subsets, 0.9865 for verification subsets, 0.9170 for test subsets and 0.8735 for the total dataset to indicate acceptable overall fitting capacity despite limited generalization performance of the network. Reliability validation outcomes are summarized in Figure 7, and consistent variation tendencies as well as minor global deviations were detected between network-predicted and experimentally measured sensory values to confirm stable and reliable network performance. The established BPNN shows strong data-simulation capacity and accurately describes the mapping links between processing parameters and sensory scores.

#### 3.3.2. Extended Parametric Simulation and Prediction of BPNN

Restrictions of RSM within limited experimental design space were overcome via extended prediction simulation of the trained BPNN model, and parallel comparison was conducted between network-optimized and response surface-optimized formulations for reliability validation. Systematic grid traversal search covering 29,791 virtual parametric combinations was implemented within parameter ranges of 30–50% sugar substitute dosage, 0.5–2.5 Xyl/IMO ratio and 4–8 h soaking time to predict corresponding sensory scores. Theoretical optimal technological parameters predicted by BPNN were determined as 41.30% sugar substitute addition (based on strawberry mass), Xyl/IMO ratio of 1.70 and soaking time of 5.50 h, with a maximum predicted sensory score of 82.62. Practical adjustment of optimized parameters to a 40% substitute dosage, 1.50 Xyl/IMO ratio and 5.5 h soaking time was completed for subsequent parallel verification trials considering industrial production feasibility.

### 3.4. Parallel Verification of RSM and BPNN Optimized Parameters

Triplicate parallel verification experiments were conducted under parametric combinations separately optimized by RSM and BPNN to validate prediction reliability of the two models. An average sensory score of 81.63 ± 0.0665 was obtained from three repeated trials under RSM-optimized conditions (45% sugar substitute dosage, Xyl/IMO ratio of 1.5, soaking time of 5.5 h), while a higher average sensory score of 82.72 ± 0.0356 was generated by samples manufactured under BPNN-optimized practical parameters (40% sugar substitute dosage, Xyl/IMO ratio of 1.50, soaking time of 5.5 h). Merely a one-point difference between the two predicted sensory values confirmed high prediction accuracy and applicability of both modeling approaches for strawberry preserve formulation optimization, yet a numerically higher sensory score and improved experimental repeatability were obtained under the BPNN-optimized technological parameters, through which final optimal processing conditions were confirmed as 40% compound sugar substitute addition, Xyl/IMO mass ratio of 1.5 and soaking time of 5.5 h.

Our parallel model comparison draws conclusions consistent with Yang [25], showing that BPNN delivers better overall performance than RSM for food-processing optimization. Previous studies have widely confirmed that BPNNs achieve higher fitting and prediction precision for nonlinear variable correlations and complex interactions. Such performance gaps originate from intrinsic modeling mechanisms of the two algorithms. Second-order polynomial fitting is adopted by RSM to ignore high-order interaction terms and introduce inherent modeling errors, while more complex multi-dimensional mapping correlations between multiple variables can be accurately captured via neural network training [26].

### 3.5. Comparative Physicochemical Characterization of Sugar-Substituted and Sucrose-Controlled Strawberry Preserves

Quantitative results of total sugar, titratable acidity, total flavonoids, total phenolics, water activity and moisture content of two groups of strawberry preserves are summarized in Table 5. Significantly lower total sugar concentrations were detected in sugar-substituted samples relative to sucrose-controlled preserves, yet all tested products satisfied the 75% upper limit of total sugar specified in the national standard GB/T 10782-2021 for candied fruits [27]. Sugar-substituted preserves show lower titratable acidity (calculated as malic acid), falling within the standard range of 0.1–0.8%, and higher contents of total flavonoids and total phenolics were simultaneously measured in sugar-substituted samples. The mean moisture content values of both sample groups met the national standard limit of below 20% [27], with slightly higher moisture detected in sugar-substituted strawberry preserves. Standard color parameters of fresh strawberry raw materials were measured as L_0_* = 40.50 ± 0.22, a_0_* = 26.12 ± 0.10 and b_0_* = 24.51 ± 0.44, and complete color measurement data of finished preserves are listed in Table 6. Slightly higher L* values yet lower a* and b* values were recorded for sugar-substituted preserves in comparison with sucrose-controlled counterparts, indicating elevated lightness alongside weakened red and yellow chromaticity in low-sugar samples.

The lower total-sugar content of sugar-substituted preserves arises from differing molecular weights and osmotic-pressure gradients produced by distinct sweetener systems [28]. Slightly elevated titratable acidity in sucrose-preserved samples may be associated with sucrose fermentation or intermediate by-products produced during thermal Maillard reactions [29]. Ultrasonic-processing-enhanced antioxidant performance together with the mild intrinsic antioxidant activity of compound sweeteners jointly help low-sugar preserves retain more total flavonoids and phenolics [30]. Furthermore, the prebiotic functional characteristics of Xyl and IMO, together with the preferential oxidative degradation of ascorbic acid during hot-air drying, may contribute to the observed retention of phenolic bioactive substances [31]. The slightly higher moisture content of sugar-substituted preserves may be associated with weaker osmotic dehydration efficiency and the relatively stronger hygroscopicity of the Xyl and IMO compound sweeteners. Distinct color differences between the two sample groups are mainly generated by varied thermal reactivity of different sweeteners. The slightly lower fruit lightness of sucrose-preserved samples may be related to the formation of dark-colored caramelization and Maillard reaction products during heating. The relatively vivid red pigmentation could also be associated with a potential protective effect of sucrose against anthocyanin degradation. In contrast, Xyl and IMO feature low thermal reactivity. They preserve higher fruit lightness but offer weaker protection for anthocyanin molecules, which causes obvious loss of red–yellow chroma [32]. Overall advantages of sugar-substituted strawberry preserves are embodied in reduced total sugar concentration and higher retention of bioactive antioxidant substances, yet further parametric adjustment is required for optimized pigment retention and moisture control performance.

## 4. Conclusions

Higher predicted sensory scores under identical optimal processing conditions verify that BPNN possesses better optimization capacity than BBD-based RSM. Confirmed optimal technological parameters for low-sugar strawberry preserves included a 40% (*w*/*w*) addition dosage of compound Xyl–IMO sugar substitute, a Xyl/IMO mass ratio of 1.5, and an osmotic soaking duration of 5.5 h. Higher contents of total phenolics and flavonoids alongside lower titratable acidity and total sugar concentrations were detected in sugar-substituted strawberry preserves relative to sucrose-controlled samples, while both product groups fully complied with national standard limits of moisture content and water activity. Stronger red–yellow pigmentation was exhibited by sucrose-preserved strawberry samples, whereas elevated lightness was obtained from low-sugar sugar-substituted products. Partially replacing sucrose with Xyl and IMO delivers clear technical benefits for reducing total sugar and preserving bioactive components in strawberry preserves. Our results validate that these optimized processing parameters are feasible for large-scale industrial production; low-sugar sugar-substituted strawberry preserves therefore hold promising application potential. Notably, the BPNN optimization strategy is compatible with conventional strawberry preserve production lines without major equipment retrofitting, which enables its potential adoption in real industrial batch production of low-sugar preserves. However, storage stability and microbial safety were not evaluated in this study. Future studies should further investigate the storage stability and microbial safety of the optimized sugar-substituted strawberry preserves.

## Figures and Tables

**Table 1:** Experimental Factors and Levels.

Level	Factor		
	Factor A: Sugar Substitute Addition/%	Factor B: Xyl/IMO Mass Ratio	Factor C: Soaking Time/h
−1	35	1:1	4
0	40	3:2	5
1	45	2:1	6

**Table 2:** Sensory Evaluation Criteria.

Sensory Attribute	Evaluation	Score/Points
	Balanced sweet–sour	15–25
Taste (Sweet–Sour)	Slightly sour or slightly sweet	5–15
	Excessively sour or sweet	0–5
	Uniform, delicate, free of defects	15–25
Texture	Moderately uniform, good softness	5–15
	Too hard or too soft, poor texture	0–5
	No off-flavor, strong strawberry aroma	15–25
Aroma	No off-flavor, mild strawberry aroma	5–15
	Off-flavor present, no strawberry aroma	0–5
	Transparent and glossy, uniform color	15–25
Color	Opaque but fairly uniform	5–15
	Opaque and uneven	0–5

**Table 3:** Experimental design and results of the Box–Behnken response surface methodology (BBD).

Run No	A: Sweetener Addition (% of Strawberry Mass)	B: Sweetener Ratio	C: Soaking Time (h)	Sensory Score (Points)
1	−1	−1	0	75.33
2	1	−1	0	78.50
3	−1	1	0	77.33
4	1	1	0	81.00
5	−1	0	−1	75.28
6	1	0	−1	77.83
7	−1	0	1	78.00
8	1	0	1	80.22
9	0	−1	−1	75.33
10	0	1	−1	76.00
11	0	−1	1	76.67
12	0	1	1	80.00
13	0	0	0	81.50
14	0	0	0	80.67
15	0	0	0	80.83
16	0	0	0	79.83
17	0	0	0	80.50

**Table 4:** Analysis of variance (ANOVA) for the regression model.

Source of Variation	Sum of Squares	df	Mean Square	F-Value	*p*-Value	Significance
Model	76.47	9	8.50	29.41	<0.0001	**
A	16.85	1	16.85	58.32	0.0001	**
B	9.03	1	9.03	31.26	0.0008	**
C	13.65	1	13.65	47.25	0.0002	**
AB	0.0625	1	0.0625	0.2163	0.6560	
AC	0.0272	1	0.0272	0.0942	0.7678	
BC	1.77	1	1.77	6.12	0.0426	*
A^2^	3.39	1	3.39	11.72	0.0111	*
B^2^	12.59	1	12.59	43.58	0.0003	**
C^2^	15.79	1	15.79	54.67	0.0002	**
Residual	2.02	7	0.2889			
Lack of fit	0.5735	3	0.1912	0.5277	0.6867	
Pure error	1.45	4	0.3622			
Total	78.49	16				

Note: ** *p* < 0.01 indicates a highly significant effect; * 0.01 ≤ *p* < 0.05 indicates a significant effect. (Sum of squares is Type III-Partial).

**Table 5:** Physicochemical properties of sugar-substituted and sucrose-sweetened strawberry preserves.

Parameter	Sugar-Substituted Preserves	Sucrose-Sweetened Preserves
Total sugar (g/100 g)	49.16 ± 2.31 ^a^	66.00 ± 2.46 ^b^
Total acidity (g/kg, as malic acid)	1.273 ± 0.25 ^a^	2.613 ± 0.16 ^b^
Total flavonoids (mg/g)	24.73 ± 0.59 ^a^	17.88 ± 0.15 ^b^
Total phenolics (mg/g)	113.83 ± 5.39 ^a^	79.67 ± 2.78 ^b^
Moisture content (%)	19.84 ± 0.38 ^a^	18.21 ± 0.53 ^b^
Water activity (a_w)	0.623 ± 0.003 ^a^	0.614 ± 0.002 ^b^

Note: Different lowercase letters within the same row indicate significant differences between treatments (*p* < 0.05, *n* = 3).

**Table 6:** Color parameters of strawberry preserves.

Color Parameters	Sugar-Substituted Preserves	Sucrose-Sweetened Preserves
L*	32.92 ± 0.77 ^a^	31.21 ± 0.85 ^a^
a*	19.35 ± 0.88 ^a^	27.55 ± 0.85 ^b^
b*	12.37 ± 0.34 ^a^	15.28 ± 0.17 ^b^
ΔE	15.83 ± 0.59 ^a^	13.17 ± 0.62 ^b^

Note: Different lowercase letters within the same row indicate significant differences between treatments (*p* < 0.05, *n* = 3).

## Data Availability

The original contributions presented in this study are included in the article. Further inquiries can be directed to the corresponding author.
